# Responding to Intergenerational Food Security and Nutrition Education Needs With Remote Programming

**DOI:** 10.1093/geroni/igab046.1554

**Published:** 2021-12-17

**Authors:** Rachel Scrivano, Shannon Jarrott, Jill Juris Naar

**Affiliations:** 1 The Ohio State University, Columbus, Ohio, United States; 2 Appalachian State University, Boone, North Carolina, United States

## Abstract

In-person intergenerational programming focused on nutrition education and healthy food access among older adults and preschool children in care settings was abandoned last year when COVID forced center closures. Food for a Long Life (FFLL), a 5-year community-based participatory research (CBPR) project, re-oriented programming in response to heightened community food insecurity and social isolation during COVID. With county Extension agents, FFLL modified and initiated new partnerships to expand food pantry services for several hundred families and deliver nutrition programming to youth (n=28) and older adult (n=130) participants in two states. In this presentation we share how the CBPR method supported adaptive programming and evaluation while continuing to advance project goals, including to promote the sustainability of an intergenerational food pantry and nutrition programming delivery after funding ends in summer 2021.

